# Isoquinoline alkaloids supplementation on performance and carcass traits of feedlot bulls

**DOI:** 10.5713/ajas.17.0868

**Published:** 2018-03-02

**Authors:** Alex Michels, Mikael Neumann, Guilherme Fernando Mattos Leão, Angela Maria Reck, Heloisa Godoi Bertagnon, Leandro Sâmia Lopes, André Martins de Souza, Leslei Caroline dos Santos, Edelmir Sílvio Stadler Júnior

**Affiliations:** 1 Department of Animal Sciences, Santa Catarina State University, Chapecó, 89.815-630, Brazil; 2 Department of Veterinary Science, University of Midwest, Guarapuava, 85040-080, Brazil; 3 Department of Animal Sciences, Federal University of Paraná, Curitiba, 80035-050, Brazil; 4 Department of Veterinary Science, Federal University of Minas Gerais, Belo Horizonte, 31270-901, Brazil

**Keywords:** Alkaloids of Benzophenanthridine and Protopine, High Energy Diet, Dry Matter Intake

## Abstract

**Objective:**

Isoquinoline Alkaloids, derived from one plant (*Macleaya cordata*) can be an alternative when it is desired to increase performance in feedlot cattle. However, results on these nutritional additives in high energy diets in ruminants are still incipient in literature. In this context, the objective of this study was to evaluate performance and carcass traits of feedlot bulls supplemented with sanguinarine, the main alkaloid presents in *Macleaya cordata* in high energy density diets.

**Methods:**

Thirty-two crossbred Angus-Nelore bulls with mean initial body weight of 365±10 kg and mean initial age of 11±3 months were used. The experiment lasted 119 days, with 14 days of adaptation and 105 experimental days. Experimental diet consisted of 85% whole corn grains and 15% protein-vitamin-mineral nucleus and supplied *ad libitum*. Treatments consisted of a control diet (CON) and a diet with sanguinarine supplementation (SAN) at a dosage of 4 g of product sufficient to provide 6 mg of sanguinarine/d. Experimental design was completely randomized.

**Results:**

Dry matter intake, average daily gain and feed conversion were similar (p>0.05) between treatments. However, SAN group animals had higher carcass yield (p = 0.045) and were more efficient in the transformation of dry matter consumed in carcass gain (p = 0.046) than CON. In addition, haptoglobin, increased throughout feedlot duration meaning high challenge for the animals due to the diet, but this behavior was similar (p>0.05) between treatments.

**Conclusion:**

Sanguinarine produced positive results in relation to carcass yield and could be used as an additive for bulls fed diets receiving high energy density diet.

## INTRODUCTION

Feedlot cattle is one of the major highlights of the Brazilian agribusiness worldwide, with the second major effective cattle population and the major export herd in the world [1]. In this economic context, to be competitive, livestock must prioritize technical and economic efficiency with the adoption of tools to optimize productivity and reduce the use of antibiotics, thus assuring better health to animals and consumers [2].

In this context, isoquinoline alkaloids, derived from one plant (*Macleaya cordata*) may become a relevant nutritional additive for beef cattle. Sanguinarine is the main alkaloid present in *Macleaya cordata* is a feed additive produced from these Papaveraceae family. These plants contain benzophenanthridine and protopine (QBA+PA), and these are known for their antimicrobial properties *in vitro*, as they inhibit the bacteria and fungi multiplication that cause gastrointestinal discomfort [3], in addition to have an anti-inflammatory function to assure a better animal health [4–8].

In high energy diets with excess content of starch, acidosis is the main metabolic disorder occurring in feedlot cattle [9]. On its turn, this disorder accounts for the remarkable change in the microbial rumen population [10] and for the increase of free rumen lipopolysaccharide, which is then released into blood current to activate an inflammatory response [11]. The ruminal acidosis also injures the rumen wall, as it can cause rumenitis and, as the ruminal pH drops, the range and frequency of ruminal movements also drop, leading to damage digestibility, nutrients absorption and, as a consequence, the feed efficiency [10]. In this scenario, sanguinarine activity as an anti-inflammatory agent may be an option to reduce the issues involved in the high energy density diets.

Therefore, this study aimed to evaluate performance and carcass traits of feedlot cattle fed high density energy diets and with sanguinarine supplementation.

## MATERIALS AND METHODS

### Local and animal care

Experiment was conducted at Guarapuava, Paraná, Brazil (25°23′02″ S, 51°29′43″ W, with an altitude of 1,098 meters), from November, 2015 till February, 2016. Guarapuava region has a humid mesothermic subtropical climate (Cfb), no dry season, cool summer and moderate winter. Guarapuava has 1,944 mm of average annual rainfall, 12.7°C of average annual minimum temperature and 23.5°C average annual maximum, and 77.9% average relative humidity.

All experimental procedures have been previously submitted to the appreciation of the Committee for Ethics in the Use of Animals for Trials (CEUA), and duly approved for implementation (Opinion n. 021/2015 dated September 11th, 2015).

### Diets and feeding

Thirty-two crossbreeds Nellore-Angus bulls sourced from a single herd, with initial average body weight (BW) of 365±10 kg and initial average age of 11±3 months were used in the experiment. The animals were dewormed and housed in 16 semi-covered confinement pens (15 m^2^), with concrete feeders, and float-regulated waterers.

Treatments consisted of: CON (without sanguinarine addition) and SAN (diet supplemented with sanguinarine). The dose used was 4 g animal/d of Sangrovit RS (Phytobiotics, Eltville, Germany), sufficient to turn it available 6 mg of sanguinarine/d, as the manufacturer’s recommendation. The additive was supplied as top-dress on the feed, aiming at assuring the intake to the animals.

The diet consisted of 85% whole corn grains and 15% protein-vitamin-mineral mix, and was supplied “*ad libitum*”. The mixture was provided as total mixed ration.

The feedlot trial had a duration of 119 days, with initial 14 days period of adaptation to the diets and to the experimental facilities, followed by five evaluation periods each lasts 21 days. Feeding management was performed twice a day, at 6:00 am and at 5:30 pm.

Adaptation protocol to the high energy diet consisted in the first four days the animals received 1.2% of BW of concentrate mixture (protein-vitamin-mineral nucleus): 15%+ whole corn grain: 85% and corn silage (*ad libitum*); as of the fifth day it was provided 1.6% of BW of the concentrate mixture and corn silage (*ad libitum*); in the tenth day it was provided 1.8% of BW and it was started a reduction of corn silage supply of 25% in relation to the previous day until the corn silage, thus turning available only the concentrate mixture in the trough as “*ad libitum*” with daily supply adjustments.

The pelletized concentrate was consisted of: soybean meal, wheat bran, malt radicle, calcitic limestone, dicalcium phosphate, urea livestock, vitamin and mineral premix, common salt, monensin sodium (75 mg/kg) and virginiamicin (75 mg/kg).

Diet samples from each treatment were collected during the experimental period (Table 1). Composite feed samples were dried in a forced-air oven at 55°C for 72 hours to obtain dry matter (DM) content, and sequentially ground in a Wiley mill using a 1 mm diameter sieve. Samples were analyzed for mineral matter, crude protein and fat [12]. Neutral detergent fiber and acid detergent fiber fractions were analyzed using thermo-stable α-amylase [13,14], total digestible nutrients were estimated [15] and calcium and phosphorus were also analyzed [16].

### Performance and behavioral test

Animals were weighted at the end of the adaptation phase and the performance evaluations were carried out every 21 days, in sequence, totaling five evaluation periods. The weight was obtained following a ten-hour fasting of solids.

Dry matter intake (DMI) was measured by the difference between amount of feed offered and orts from the previous day. DMI in % of the live weight (DMI BW) was expressed at by the ratio between DMI and the average BW in %. Average daily gain (ADG) was calculated by the difference between BW at the end and at the beginning of experimental period divided by the days evaluated. Feed conversion (FC) was obtained by the ratio between DMI and ADG.

A behavioral analysis was performed for 48 continuous hours in the middle of the experiment. Observations were performed by 9 individuals per shift in a 6-hour rotation cycle. Readings were collected in every 3 min. Behavioral data included time spent eating (hours per day and number of times per day), drinking water (hours per day and number of times per day), ruminating (hours per day), and resting (hours per day) [17]. The events of xylophagy, solid and liquid excretions per day were also evaluated. Xylophagy may be described as a behavior in which the animal nibbles wood to stimulate salivation as a response to a low ruminal pH [18].

### Blood analysis

Besides, to indicate parameter for inflammatory response, serum haptoglobin was measured in five periods, on day 1, 21, 42, 63, and 105 during feedlot. For this purpose, blood samples were collected by venipuncture of jugular vein via evacuated serum tubes (BD Vacutainer, Franklin Lakes, NJ, USA) containing clot activator. Serum were obtained from samples by centrifugation (3,500 rpm during 15 min) and frozen at −20°C until the analysis. Haptoglobin has been measured by ELISA technique, using commercial kits (Finetest, Wuhan, China).

### Carcass traits

At the end of the experiment, after fasting from solids for 10 hours, the bovines were weight. The slaughter was conducted according to inspection norms, with animals being rendered insensible by cerebral concussion followed of section of the jugular vein, leather removal and evisceration [19].

The subcutaneous fat thickness was measured at the *Longissimus dorsi*, between rib 12 and 13, using a caliper [20]. At the carcasses, five development values were taken: carcass length (distance between the cranial medial edge of the pubis bone and the cranial medial edge of the first rib); leg length (distance between the cranial medial edge of the pubis and the radiocarpal joint); arm length (distance between the olecranon tuberosity and the radiocarpal joint); arm perimeter (obtained by surrounding it with a metric tape) in the median region of the arm, and the thigh thickness (measured with the help of a compass, perpendicular to the carcass length, taking the greatest distance between the cut that separates the two half-carcasses and the lateral muscles of the thigh) [21].

Estimated efficiency parameters, carcass daily gain (CDG) and carcass feed efficiency (CG:F) were calculated using ADG, DMI, and hot carcass weight (HCW). Carcass total weight gain was calculated as the difference between HCW and initial carcass weight (iCW), which was estimated considering an initial carcass yield of 50% (iCW = initial BW×0.50). The CDG was calculated for the 119 days of confinement (CDG = Total carcass gain÷119). Efficiency of transformation of gain in carcass (CDG/ADG) was obtained by a ratio between CDG and ADG. And CG:F was represented by the ratio between CDG and DMI (CG:F = DMI÷CDG).

At the time of slaughtering, characterization of non-carcass components was also made by collecting the weight of the following components: head, tongue, tail, leather and paws (referred to as external components); and heart, kidneys, liver, lungs, spleen, empty rumen-reticulum, full rumen-reticulum, empty abomasum, full omasum and full intestines (referred as vital organs) [21].

### Statistical analysis

Experimental design was totally randomized, comprising two treatments with eight repetitions, where each repetition corresponded to one bay with two animals. Data collected for each variable were submitted for analysis of variance at 5% significance, using the MIXED procedure of statistical program SAS (SAS Inst. Inc., Cary, NC, USA) The analysis of each variable followed the statistical model: Y_ij_ = μ+S_i_+E_ij_; Where: Y_ij_ = dependent variables; μ = general average of all the observations; S_i_ = sanguinarine effect of order “i”, being 1 = CON diet and 2 = SAN diet; and E_ij_ = residual random effect.

## RESULTS AND DISCUSSION

In performance evaluation, treatments were similar (p>0.05) for ADG, DMI (kg), DMI in % of BW and FC during feedlot periods or in the feedlot total period (Table 2). In high energy diets, ruminal pH tends to be lower than 6.0 due to the higher amount of fermentable carbohydrates, which can lead to ruminal acidosis [9]. This issue may justify the lack of positive results for SAN, as there is a relationship between average pH and the role of the isoquinoline alkaloids [22]. In this study, the authors concluded that pH lower than 6.0 implies in a reduction in sanguinarine capacity to act positively in the volatile fat acids production and to model the rumen flora.

Furthermore, in high energy density diets, DMI tends to be ruled by metabolic ways [23], and sanguinarine was not able to change this parameter. And obviously, these results could not impact in FC. Although performance was not modified, SAN presented lower CG: F (p = 0.046), what suggests that animals need lower volume of DM consumed to produce carcass (Table 3). This may be the result of the increase in efficiency in dietary energy [24] and protein digestion [25] use in feedlot cattle with SAN supplementation.

**Figure 1:**
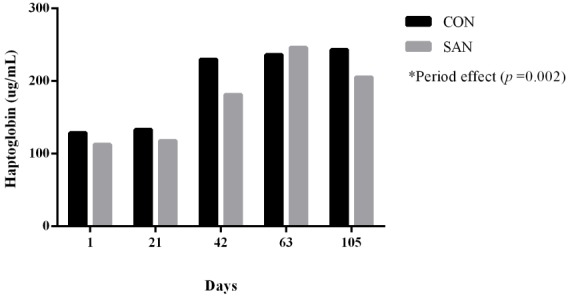
Haptoglobin levels of feedlot bulls supplemented (SAN) or not (CON) in high energy diets.

As regards haptoglobin, there were an increase of this pro-inflammatory protein during the feedlot period (Figure 1). In healthy animals, these acute phase protein concentrations are between 100 to 200 μg/mL [26–28]. These results suggest that animals presented signs of an inflammatory process and it could be justified by the subclinical ruminal acidosis provided by the diet. However, SAN was not enough to control inflammatory condition, as the haptoglobin levels were similar (p> 0.05) between the treatments. Even though, some studies have found lower neutrophil infiltration in the ruminal tissue with SAN [29].

Furthermore, SAN did not modulate the ingestive behavior (Table 4). Presently, literature is scarce on ingestive behavior studies that used sanguinarine in beef cattle nutrition. However, few studies with sheep did not demonstrate differences with sanguinarine as regards the control treatment [29].

However, xylophagy was superior (p = 0.0028) for the SAN group. Ruminants consuming diets without forages, have a tendency to drop the ruminal pH, tend to usually practice xylophagy, which may be explained by their behavior to physically stimulate saliva production [30]. An explanation for the xylophagy increase in animals feed with SAN may be linked to the greater diet digestibility, thus increasing volatile fat acids production in the rumen and inducing acidosis [31].

As regards the carcass traits, it was noted a greater carcass yield (p = 0.045) for all the SAN group animals (Table 5). This is a relevant finding, because carcass yield is important for economically purposes [32]. Besides, these results are not previously reported in literature [29]. The other carcass parameters, in turn, were not changed by SAN.

As regards the carcass non integrating yield components, no difference (p>0.05) was found between the treatments (Table 6). The size and weight of vital organs are associated with the metabolic activity and with the slaughter higher energy requirements [33,34]. This way, it is noted that the inclusion of sanguinarine did not change the animals’ metabolic activity and the slaughter requirements, in agreement with others studies [29].

In turn, leather weight was lower (p = 0.018) for the SAN group. This may be justified by the relationship existing between leather weight and carcass yield [35]. That is, lighter leathers may positively affect this parameter, duly evidenced by the higher carcass yield for animals supplemented with sanguinarine.

Sanguinarine used in high energy diets in feedlots provided improvements in efficiency to transform DM into carcass and in the carcass yield. More studies are suggested to confirm these good results.

## Figures and Tables

**Table 1:** Chemical composition of feeds and experimental diet

Parameters	Whole corn grain	Concentrate1)	Experimental diet2)
Dry matter content (% of natural matter)	90.0	90.2	90.1
Crude protein (% of DM)	7.9	42.2	13.0
Mineral matter (% of DM)	0.76	16.3	3.1
Fat (% of DM)	3.4	2.6	3.3
Neutral detergent fiber (% of DM)	17.1	24.6	18.3
Acid detergent fiber (% of DM)	5.9	12.3	6.9
Total digestible nutrients (% of DM)	83.7	69.7	81.6
Calcium (% of DM)	0.03	2.8	0.44
Phosphorus (% of DM)	0.25	1.1	0.38

DM, dry matter; CP, crude protein; NDF, neutral detergent fiber; ADF, acid detergent fiber; TDN, total digestible nutrients.

1) Concentrate composition: DM, 90.22%; CP, 42.23%; NDF, 24.61%; ADF, 12.28%; inorganic matter, 16.31%; fat, 2.95; TDN, 69.70; phosphorus, 1.11%; calcium, 2.77%; monensin, 75 mg/kg; and virginiamycin, 75 mg/kg.

2) Diet composition: 85% of whole corn grain and 15% concentrate.

**Table 2:** Performance of feedlot bulls supplemented (SAN) or not (CON) in high energy diet

Parameter	Experimental diet		Mean	SEM	p value

	SAN	CON			
ADG (kg/d)					
0–21 days	1.15	1.12	1.14	0.06	0.869
0–42 days	1.19	1.22	1.20	0.06	0.853
0–63 days	1.21	1.30	1.26	0.07	0.537
0–84 days	1.24	1.33	1.29	0.07	0.531
0–105 days	1.25	1.32	1.28	0.06	0.561
DMI (kg/d)					
0–21 days	6.82	7.09	6.96	0.22	0.560
0–42 days	6.93	7.21	7.07	0.24	0.565
0–63 days	6.85	7.32	7.09	0.25	0.362
0–84 days	6.96	7.55	7.25	0.26	0.278
0–105 days	7.09	7.72	7.41	0.27	0.275
DMI (% BW)					
0–21 days	1.75	1.80	1.78	0.05	0.583
0–42 days	1.72	1.77	1.74	0.05	0.601
0–63 days	1.64	1.73	1.69	0.04	0.363
0–84 days	1.61	1.73	1.67	0.04	0.235
0–105 days	1.60	1.71	1.65	0.04	0.222
FC (DMI/ADG)					
0–21 days	6.53	6.99	6.76	0.35	0.828
0–42 days	6.30	6.25	6.27	0.29	0.894
0–63 days	5.77	5.81	5.79	0.26	0.926
0–84 days	5.72	5.78	5.75	0.21	0.889
0–105 days	5.74	5.94	5.84	0.17	0.590

SEM, standard error mean; ADG, average daily gain; DMI, dry matter intake; BW, body weight; FC, feed conversion.

**Table 3:** Carcass daily gain (CDG), Efficiency of CDG in relation of ADG (CDG/ADG) and carcass feed efficiency (CG:F) of feedlot bulls supplemented (SAN) or not (CON) in high energy diet

Parameter	Experimental diet		Mean	SEM	p value

	SAN	CON			
CDG (kg/d)	0.89	0.90	0.90	0.04	0.868
CDG/ADG (%)	73.2^a^	68.2^b^	70.7	1.14	0.045
CG:F (DMI/carcass, kg)	8.1^b^	8.7^a^	8.37	0.26	0.046

SEM, standard error mean; ADG, average daily gain.

Averages followed by different superscript letters (^a,b^) in the same row, are significant different by Tukey test (p≤0.05).

**Table 4:** Ingestive behavior of feedlot bulls supplemented (SAN) or not (CON) in high energy diets

Parameter	Experimental diet		Mean	SEM	p value

	SAN	CON			
	--------------- Hours/d --------------				
Feed intake	2.05	2.03	2.03	0.11	0.9314
Water intake	0.28	0.25	0.25	0.01	0.6140
Chewing	0.83	0.97	0.97	0.05	0.3562
Resting	20.9	20.8	20.8	0.24	0.6671
	--------- Number of times/d ---------				
Feed intake	13.0	13.41	13.41	0.65	0.5417
Water intake	6.9	6.59	6.59	0.33	0.6784
Liquid excretion	3.6	3.75	3.75	0.20	0.6399
Solid excretion	2.8	2.81	2.81	0.15	0.9999
Xylophagy	5.3	3.94	3.94	0.19	0.0028

SEM, standard error mean.

**Table 5:** Carcass traits of feedlot bulls supplemented (SAN) or not (CON) in high energy diets

Parameter	Experimental diet		Mean	SEM	p value

	SAN	CON			
Hot carcass (kg)	287.1	287.4	287.2	5.61	0.978
Carcass yield (%)	56.4^a^	55.4^b^	55.9	0.28	0.045
Fat thickness (mm)	5.6	5.9	5.8	0.37	0.616
Carcass length (cm)	126.7	130.4	128.6	0.74	0.055
Thigh thickness (cm)	21.1	20.4	20.8	0.74	0.620
Arm length (cm)	37.0	37.0	37.0	0.22	0.891
Arm perimeter (cm)	39.0	39.5	39.3	0.46	0.581

SEM, standard error mean.

Averages followed by different superscript letters (^a,b^) in the same row, are significant different by Tukey test (p≤0.05).

**Table 6:** Non-carcass components of feedlot bulls supplemented (SAN) or not (CON) in high energy diets

Parameter	Experimental diet		Mean	SEM	p value

	SAN	CON			
Vital organs	------- % of body weight ------				
Heart	0.34	0.33	0.33	0.01	0.378
Liver	1.10	1.16	1.13	0.03	0.434
Lungs	0.89	0.98	0.93	0.02	0.061
Kidneys	0.19	0.19	0.19	0.01	0.926
Spleen	0.30	0.33	0.32	0.01	0.194
Full rumen/reticulum	6.88	7.78	7.33	0.31	0.171
Empty rumen/reticulum	2.11	2.22	2.17	0.05	0.273
Omasum	0.74	0.69	0.72	0.02	0.282
Abomasum	0.82	0.81	0.81	0.02	0.979
Full intestines	3.32	3.30	3.31	0.07	0.908
External components					
Head	1.90	1.96	1.93	0.03	0.236
Tongue	0.15	0.16	0.15	0.00	0.326
Leather	8.88^b^	9.63^a^	9.26	0.14	0.018
Tail	0.22	0.25	0.23	0.01	0.078
Paws	1.99	2.20	2.09	0.06	0.091

SEM, standard error mean.

Averages followed by different superscript letters (^a,b^) in the same row, are significant different by Tukey test (p≤0.05).
